# Comparative Renal Outcomes and Effectiveness of Non-Vitamin K Antagonist Oral Anticoagulants Versus Warfarin in Nonvalvular Atrial Fibrillation: Insights from Real-World Data

**DOI:** 10.3390/medicina62030532

**Published:** 2026-03-13

**Authors:** Karatpetch Tongkate, Thoranis Chantrarat, Pornwalai Boonmuang, Weerayuth Saelim, Narisa Ruenroengbun, Junporn Kongwatcharapong

**Affiliations:** 1 The College of Pharmacotherapy of Thailand, Nonthaburi 11000, Thailand; karatpetch@eau.ac.th; 2 Pharmaceutical Care Division, School of Pharmacy, Eastern Asia University, Pathum Thani 12110, Thailand; 3 Division of Cardiology, Department of Medicine, Phramongkutklao Hospital, Bangkok 10400, Thailand; thoranis@gmail.com; 4 Division of Pharmaceutical Care, Faculty of Pharmacy, Silpakorn University, Nakhon Pathom 73000, Thailand; saelim_w6@su.ac.th (W.S.); ruenroengbun_n@su.ac.th (N.R.); 5 Pharmacy Department, Faculty of Medicine Siriraj Hospital, Mahidol University, Bangkok 10700, Thailand; hung_rx@hotmail.com

**Keywords:** non-vitamin K antagonist, warfarin, renal outcomes, atrial fibrillation

## Abstract

*Background and Objectives*: While non-vitamin K antagonist oral anticoagulants (NOACs) show better renal preservation than warfarin in nonvalvular atrial fibrillation (NVAF) patients, real-world evidence within Asian populations remains limited. This study compared the renal outcomes between NOACs and warfarin in Thai patients with NVAF. *Materials and Methods*: A retrospective cohort study was conducted among NVAF patients who received either NOACs or warfarin from two university hospitals in Thailand from January 2015 to December 2019. The primary outcome was a ≥30% decline in the estimated glomerular filtration rate (eGFR) with a doubling of the serum creatinine (SCr), while acute kidney injury (AKI) and incidence rate of stroke and systemic embolism event (SEE) were secondary outcomes. All outcomes of each NOAC versus the warfarin group were analyzed using Cox proportional hazards regression. *Results*: A total of 1456 patients were enrolled. During a follow-up period of 24 months, NOACs were associated with a lower risk of a ≥30% decline in the eGFR than warfarin after inverse probability of treatment weighting (IPTW) and multivariable adjustment (adjusted hazard ratio (aHR) of 0.67, 95% confidence interval [CI] 0.45–1.00, *p* = 0.050). No significant differences were observed between NOACs and warfarin regarding the doubling of SCr (aHR 0.64, 95% CI 0.24–1.72, *p* = 0.373) or AKI (aHR 0.69, 95% CI 0.41–1.17, *p* = 0.169), although a trend toward a lower risk was noted in the NOAC group. Similarly, a trend toward a lower risk of the incidence rate of ischemic stroke and SEE were observed in the NOAC group (aHR 0.49, 95% CI 0.22–1.10, *p* = 0.084). *Conclusions*: In real-world data, NOACs may be associated with a lower eGFR decline and lower doubling of SCr and AKI than warfarin. Additionally, NOACs may reduce the risk of ischemic stroke and SEE, supporting their potential benefit over warfarin in both renal and thromboembolic outcomes.

## 1. Introduction

Currently, the prevalence of atrial fibrillation (AF) is approximately 1–4% of the general population worldwide and ranges between 2.8 and 3.46% in Thailand [1,2,3,4,5,6]. Ischemic stroke is a major complication of AF that leads to high economic costs to patients and healthcare systems [5,7,8]. Oral anticoagulants serve as the cornerstone of treatment that reduces the risk of ischemic stroke and death. Clinical practice guidelines recommend vitamin K antagonists (VKAs) and non-vitamin K antagonist oral anticoagulants (NOACs) including dabigatran, rivaroxaban, apixaban and edoxaban for stroke prevention [5,9]. Clinical trials have revealed that, in terms of efficacy and safety, NOACs provide better net clinical benefits than warfarin in patients with nonvalvular AF (NVAF) [10,11,12,13]. Current international guidelines, including the European Society of Cardiology (ESC) [5] and the American Heart Association/American College of Cardiology (AHA/ACC) [9], recommend NOACs as the preferred first-line anticoagulation therapy over warfarin for stroke prevention in most NVAF patients due to their demonstrated greater effectiveness and safety compared with warfarin. Similarly, the Thai Heart Association guidelines [14] align with these international standards, also recommending NOACs as the first-line treatment for Thai patients with NVAF. However, the utilization of NOACs in real-world clinical practice in Thailand may be influenced by economic factors and healthcare reimbursement policies, leading to continued widespread use of warfarin. Warfarin has long been prescribed as the preferred option for AF patients with end-stage renal disease (ESRD) with or without dialysis, and limited data are available for NOACs [5,9]. However, warfarin can cause acute kidney injury (AKI) associated with warfarin-related nephropathy (WRN) [15,16]. In addition, warfarin inhibits the vitamin K-dependent protein matrix gamma-carboxyglutamic acid, which translates to renovascular calcification [17,18]. Conversely, NOACs are renoprotected by the protease-activated receptor-mediated inflammatory pathway (PAR-mediated inflammatory pathway) [19,20,21]. Pieces of emerging evidence suggest that NOACs were associated with adverse renal outcome rates lower than warfarin in AF patients with chronic kidney disease (CKD) in terms of the decline in the estimated glomerular filtration rate (eGFR), doubling of serum creatinine (SCr) and AKI [21,22,23,24,25,26]. This study was conducted primarily to compare warfarin and NOACs in terms of renal outcomes including the ≥30% decline in eGFR, doubling of SCr and AKI occurrence among NVAF patients. Although efficacy outcomes were not the primary focus, the clinical response to therapy was also collected to provide a more comprehensive evaluation of the overall benefit–risk balance, considering both safety and efficacy outcomes are crucial for a balanced assessment of their clinical utility.

## 2. Materials and Methods

This study was a multi-center, retrospective cohort study among NVAF patients who received either NOACs or warfarin from January 2015 to December 2019 in tertiary-care university hospitals in Bangkok, Thailand.

All patients were diagnosed with NVAF and enrolled in accordance with the following inclusion criteria: age ≥ 18 years, new user of oral anticoagulants (warfarin, dabigatran, rivaroxaban, apixaban or edoxaban), received oral anticoagulant treatment for at least three months and regularly followed-up at the study site. Patients who were excluded from this study comprised those with mechanical valve replacement or moderate-to-severe mitral stenosis, eGFR ≤ 15 mL/min/1.73 m^2^ or those requiring dialysis, with moderate-to-severe hepatic impairment (Child–Pugh B or Child–Pugh C), pregnant or lactating, and those with the absence of SCr or eGFR at baseline before the initiation of oral anticoagulants.

The data on all patient characteristics were extracted from the hospital databases after the study population had been identified. Patients were included and categorized into two groups as follows: (1) received warfarin and (2) received NOACs (dabigatran, rivaroxaban, apixaban or edoxaban). The key characteristic data of patients included age, gender, body weight, co-medication, comorbidities and biomarkers (e.g., SCr, eGFR and hemoglobin A1c (HbA1c)).

The primary outcome was a ≥30% decline in the eGFR, which was calculated using the Chronic Kidney Disease Epidemiology Collaboration (CKD-EPI) equation. The secondary outcomes included the doubling of SCr and AKI. In addition, the incidence rate of stroke and systemic embolism event (SEE) were reported. The ≥30% decline in the eGFR was defined in accordance with the National Kidney Foundation and the US Food and Drug Administration, which concluded that a 30% to 40% decline in the eGFR over 2–3 years is an acceptable surrogate endpoint in a clinical trial [27]. The doubling of SCr was an endpoint for clinical trials of kidney disease progression accepted by the US Food and Drug Administration [27], defined as the first occurrence of a doubling from the baseline value occurring at any time during the 2-year follow-up period. AKI was defined by the Kidney Disease Improving Global Outcomes as an increase in SCr to ≥1.5 times above baseline value, occurring within a 7-day window during the follow-up period [28]. The primary and secondary outcomes were obtained by collecting data at 3, 6, 9 and 12 months and any time for 2 years and compared with the baseline. The censoring of data was performed when a patient was lost to follow-up or at the end of the study period.

### Statistical Analysis

Descriptive variables (e.g., gender and comorbidities) were analyzed as frequencies and percentages. Continuous variables were tested for normality via the Kolmogorov–Smirnov test and reported as means and standard deviations (SD) or median (interquartile range; IQR). Student’s *t*-test or Mann–Whitney U test were utilized for continuous variables. Inverse probability of treatment weighting (IPTW) based on propensity scores was used to balance covariates. The propensity scores were analyzed using a logistic regression model that included all the baseline characteristics. The balance of baseline characteristics between the two groups was assessed using standardized mean differences (SMD), with a threshold of <0.1 indicating an optimal balance. For the primary and secondary outcomes, Cox proportional hazards regression was used to estimate the adjusted hazard ratios (aHRs) and 95% confidence intervals (CIs). The global test indicated a violation of the proportional hazards’ assumption, while *p* > 0.05 was considered to satisfy the assumption. A doubly robust approach was employed to minimize residual confounding, and any variables that remained imbalanced after weighting (SMD ≥ 0.1) were additionally adjusted within the weighted Cox model. Additionally, multivariable logistic regression analysis was performed as an exploratory analysis to identify baseline clinical factors independently associated with the occurrence of the ≥30% eGFR decline within 24 months. Variables with *p* < 0.2 in univariate analysis were entered into the multivariable model. A two-sided *p* < 0.05 was considered statistically significant. All data were recorded in Excel (Microsoft Office Professional Plus, 2020) and analyzed using SPSS Statistics for Windows, Version 27.0 (IBM Corp, Armonk, NY, USA).

## 3. Results

**Figure 1:**
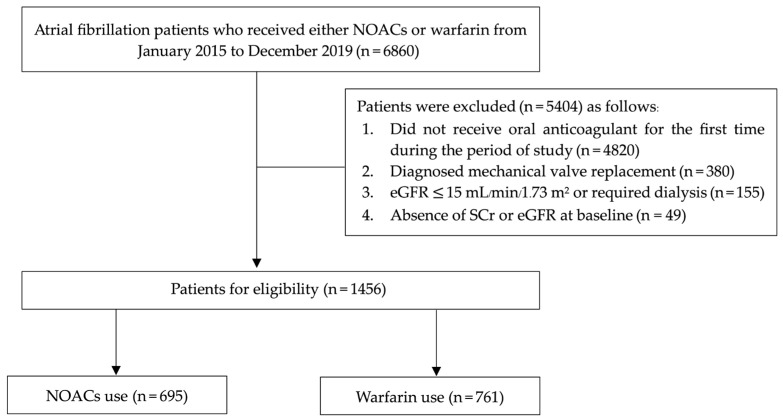
Details of patient flow.

A total of 6860 AF patients were identified from the computerized hospital database during the study period. Exactly 5404 patients were excluded due to the following: treatment with oral anticoagulants not initiated in hospital settings (*n* = 4820), mechanical valve replacement (*n* = 380), eGFR ≤ 15 mL/min/1.73 m^2^ or dialysis requirement (*n* = 155) and missing data on SCr or eGFR at baseline before the start of oral anticoagulant treatment (*n* = 49). In total, 1456 patients with NVAF received oral anticoagulants, with 761 patients belonging to the warfarin group and 695 to the NOAC group (181 patients for dabigatran, 249 patients for rivaroxaban, 217 patients for apixaban and 48 patients for edoxaban). Details of the patient flow are depicted in Figure 1.

The baseline characteristics of all patients are shown in Table 1. Before IPTW, significant baseline imbalances existed between the two groups. Compared to the warfarin group, the NOAC group was more likely to be male (59.7% vs. 53.0%, *p* = 0.009), had higher median body weight (67.5 kg vs. 64 kg, *p* < 0.001), and presented with a higher CHA_2_DS_2_-VASc score (median 4 vs. 3, *p* = 0.002). Additionally, the use of SGLT-2 inhibitors was significantly more frequent in the NOAC group (4.9% vs. 0.8%, *p* < 0.001). Conversely, the warfarin group presented with a higher HASBLED score (median 2 vs. 1, *p* < 0.001), had a higher prevalence of heart failure (27.1% vs. 15.5%, *p* = 0.002), and the use of beta-blockers was more frequent (81.1% vs. 75.1%, *p* = 0.006). Furthermore, the warfarin patients had median levels of HbA1c (6.1% vs. 5.9%, *p* = 0.024) and uric acid (6.6 mg/dL vs. 6.2 mg/dL, *p* = 0.040) which were significantly higher in the warfarin group. After IPTW, the baseline characteristics were well-balanced between the two treatment groups.

**Figure 2:**
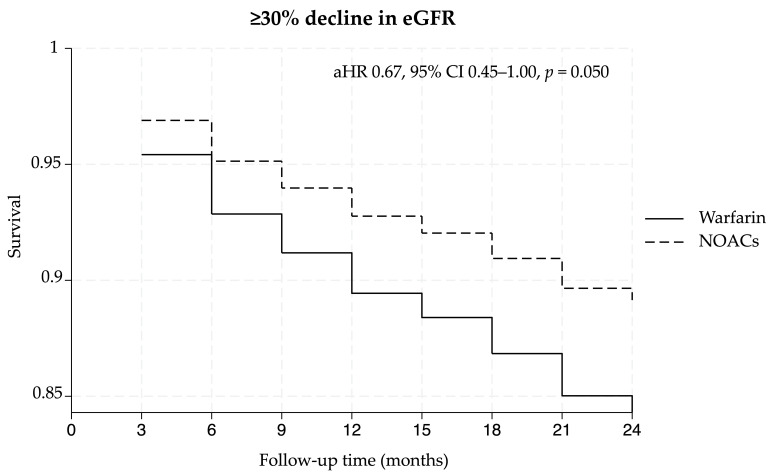
Kaplan–Meier curve of ≥30% decline in eGFR in the comparison of warfarin with NOACs. Abbreviations: NOACs, non-vitamin antagonist oral anticoagulants; aHR, adjusted hazard ratio; 95% CI, 95% confidence interval.

**Figure 3:**
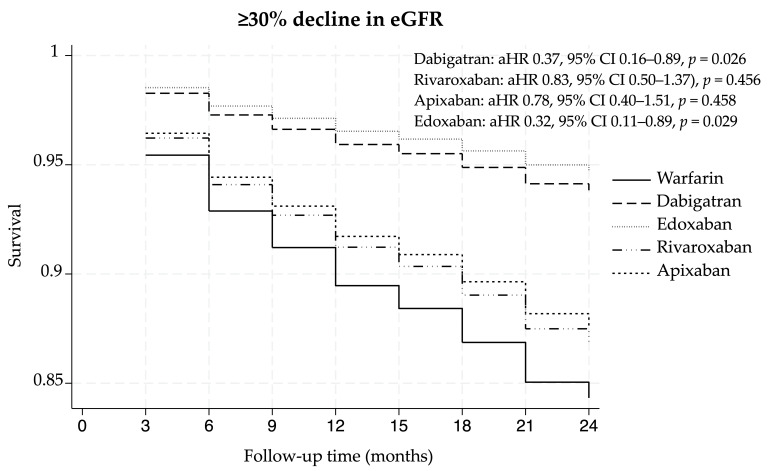
Kaplan–Meier curve of ≥30% decline in eGFR in the comparison of warfarin with each NOAC. Abbreviations: NOACs, non-vitamin antagonist oral anticoagulants; aHR, adjusted hazard ratio; 95% CI, 95% confidence interval.

During the follow-up period, a ≥30% decline in the eGFR occurred in 98 and 143 patients who received NOACs (14.1%) and warfarin (18.8%), respectively. Compared with the warfarin group, the NOAC group was associated with a lower risk of a ≥30% decline in the eGFR, with an aHR of 0.67 (95% CI 0.45–1.00, *p* = 0.050) after adjusting for all covariates using the doubly robust approach. In the subgroup analysis of individual NOACs, both dabigatran (aHR 0.37, 95% CI 0.16–0.89, *p* = 0.026) and edoxaban (aHR 0.32, 95% CI 0.11–0.89, *p* = 0.029) were associated with a significantly lower risk of a ≥30% decline in the eGFR compared to warfarin. However, a trend toward a lower risk of eGFR decline was observed in patients who received rivaroxaban and apixaban. Details about the primary outcome are shown in Table 2. The Kaplan–Meier curve of the ≥30% decline in the eGFR was obtained during the comparison of warfarin with NOACs and each NOAC (Figure 2 and Figure 3). As shown in Figure 2, the Kaplan–Meier curve of ≥30% decline in the eGFR showed the early separation in the follow-up period, with the NOAC group consistently maintaining a higher survival rate than the warfarin group. Additionally, as shown in Figure 3, the curves for dabigatran and edoxaban remained the highest among all groups.

For the secondary outcomes, the doubling of SCr and AKI occurred in 16 (2.10%) and 12 (1.73%) patients who used warfarin and NOACs, respectively. After IPTW and multivariable adjustment, NOACs were associated with no significant reduction in the doubling of SCr (aHR 0.64, 95% CI 0.24–1.72, *p* = 0.373) and AKI (aHR 0.69, 95% CI 0.41–1.17, *p* = 0.169) compared to warfarin. However, a trend toward a lower risk of secondary outcomes was observed in the NOAC group. When analyzing individual NOACs, no significant differences were observed for these outcomes across the subgroups. The doubling of SCr was notably absent among dabigatran and edoxaban groups (no events). As a result, the hazard ratios for these subgroups were not calculable. Details on the secondary outcomes are provided in Table 3.

For efficacy outcomes, the incidence rate of ischemic stroke and SEE was 1.87 per 100 person-year and 3.02 per 100 person-year in the NOAC and warfarin group, respectively. Although the initial analysis showed a significant risk reduction (Unadjusted HR 0.45, 95% CI 0.24–0.85, *p* = 0.015), but the result showed no significant difference after IPTW and multivariable adjustment (aHR 0.49, 95% CI 0.22–1.10, *p* = 0.084). Moreover, a trend toward a lower risk of the incidence rate of ischemic stroke and SEE were observed in the NOAC group. The detail of efficacy outcomes are shown in Table 4.

Multivariable logistic regression identified clinical factors that served as independent predictors of the ≥30% decline in the eGFR (Table 5). NOAC use (OR 0.70, 95% CI 0.52–0.93, *p* = 0.014) was associated with a low risk of ≥30% decline in the eGFR, whereas age ≥ 75 years (OR 1.72, 95% CI 1.28–2.32, *p* < 0.001), hypertension (OR 1.82, 95% CI 1.11–2.99, *p* = 0.018) and diabetes mellitus (OR 1.59, 95% CI 1.18–2.13, *p* = 0.002) were associated with a high risk of the ≥30% decline in the eGFR.

## 4. Discussion

This study has presented real-world evidence comparing the safety profile of NOACs and warfarin in terms of renal outcomes in Thai patients with NVAF. The principal finding of this study was that NOACs were associated with a lower risk of the ≥30% decline in eGFR compared with warfarin. However, the association reached borderline statistical significance after doubly robust adjustment. This study’s findings showed a similar direction of association to those reported by Yao et al. [21], Trevisan M et al. [29], and Shahzada et al. [30], with NOACs associated with a lower risk of ≥30% decline in eGFR compared with warfarin. However, unlike those studies, the association in this study’s cohort did not reach statistical significance after doubly robust adjustment. In contrast, Chantarat T. et al. [26] reported no significant association between NOAC use and the ≥30% decline in eGFR after multivariable adjustment (HR 1.12, 95% CI 0.67–1.87; *p* = 0.678).

In subgroup analysis of individual NOACs compared with warfarin, dabigatran and edoxaban were associated with a significantly lower risk of ≥30% decline in eGFR. The significant association observed with dabigatran was consistent with findings reported by Shahzada TS et al. [30]. In contrast, evidence of edoxaban and renal outcomes remains limited, as prior studies have not evaluated its association across all renal outcomes [21,26,29,30]. Although the observed association was statistically significant, the relatively small sample size and event numbers warrant cautious interpretation. Rivaroxaban and apixaban showed non-significant trends toward benefit. Notably, patients receiving rivaroxaban and apixaban had slightly lower median baseline eGFR (62.44 mL/min/1.73 m^2^ and 53.95 mL/min/1.73 m^2^, respectively) compared with those receiving warfarin (63.87 mL/min/1.73 m^2^). In addition, those patients were less likely to require RAAS inhibitors (50.6%, 47.9% and 51.8%, respectively), which are known to delay CKD progression. Such baseline differences in renal function and concomitant nephroprotective therapy may have influenced renal outcomes [31,32,33].

In this study, no statistically significant differences were observed between NOACs and warfarin for the secondary renal outcomes of the doubling of SCr or AKI. However, a trend toward lower risk of secondary outcomes was observed in the NOAC group. These findings differ from several previous studies that reported significant reductions in those outcomes with NOACs compared with warfarin [21,29,30]. In subgroup analysis of individual NOACs which compared warfarin, rivaroxaban trended to increase the risk of the doubling of SCr. This contrasts with findings from Yao X et al. [21], González Pérez A et al. [34], and Shahzada TS et al. [30], who reported significant reductions in doubling of SCr among rivaroxaban users. One possible explanation for this discrepancy may relate to dosing patterns in this study. A potential possibility was the inappropriate dose administered to more than half of the population of rivaroxaban users. Most of those patients received an underdose of rivaroxaban. Therefore, the dose should be adjusted appropriately in accordance with the dose reduction criteria based on renal function. In the subgroup analysis of individual NOACs compared with warfarin, both dabigatran and rivaroxaban showed no significantly reduced risk of AKI. In contrast, Yao X et al., [21] reported significantly lower risks of AKI among users of both dabigatran and rivaroxaban compared with warfarin. Differences in patient characteristics and event rates may partly explain this discrepancy.

Although this study found a significant association for the primary outcome (≥30% decline in eGFR), no significant differences were observed for doubling of SCr or AKI. This may be due to the lower number of events for these secondary outcomes, which limited the statistical power to detect differences. Moreover, doubling of SCr and AKI reflect more severe or acute kidney injury, while a ≥30% decline in eGFR may indicate earlier and more gradual deterioration in renal function [27]. Therefore, these outcomes may not occur simultaneously.

This study clearly demonstrated the TTR in the warfarin group, whereas previous studies comparing NOACs and warfarin in terms of renal outcomes did not report TTR data. However, the TTR in this study (46.3%) remained far lower than that recommended by the ESC guideline for the management of AF, which suggests a TTR ≥ 70% for warfarin users [5]. Poor anticoagulation control in this study was presented by subtherapeutic and supratherapeutic INR that potentially contributed to worsen renal function. This is just this study team’s hypothesis.

Beyond their anticoagulant properties, NOACs may favorably affect renal function compared with vitamin K antagonists. Several hypotheses have been proposed to explain the mechanisms underlying the association between NOACs and renal outcomes. Experimental studies have suggested that inhibition of PAR signaling may be associated with modulation of inflammatory and fibrotic pathways, oxidative stress, and endothelial function [19,20,21,35]. Conversely, warfarin has been associated with WRN, mediated through obstructive tubular red blood cell casts and glomerular hemorrhage [15,16,17,18]. However, these mechanistic pathways were not directly evaluated in the present study and should therefore be interpreted as this study team’s hypothesis.

Regarding efficacy outcomes, this study found that the NOAC group had a lower incidence rate of ischemic stroke and SEE compared to the warfarin group. Although the unadjusted analysis suggested a significant 55% relative risk reduction, this statistical significance was attenuated after applying doubly robust IPTW adjustment. Nevertheless, a consistent numerical trend toward reduced thromboembolic risk remained evident in the NOAC group. The direction and magnitude of effect were generally aligned with those reported in randomized controlled trials, e.g., RE-LY [10], ROCKET-AF [11], ARISTOTLE [12] and ENGAGE AF-TIMI 48 trials [13], as well as previous real-world studies from Asia [22,36,37]. However, statistical significance was not maintained in this study’s adjusted model. However, the TTR in this study was 46.3%, which was lower than the TTR were recommended by clinical practice guideline [5]. This finding indicates the risk of poorly controlled INR which leads to thrombotic events, especially stroke in NVAF patients. Furthermore, pharmacogenetic variability may contribute to interindividual differences in the efficacy and safety of oral anticoagulants. Genetic variants affecting drug activation, transport, and metabolism have been associated with altered systemic exposure to both dabigatran and factor Xa inhibitors. For example, polymorphisms in CES1 have been linked to variability in dabigatran plasma concentrations, while variants in ABCB1 and CYP3A4/5 may influence the pharmacokinetics of factor Xa inhibitors [38,39,40]. As evidence specific to Thai patients remains limited, such variability may represent an additional biological factor influencing thromboembolic and bleeding outcomes and warrants further investigation. In addition, potential drug–drug interactions are especially important, as the drugs are potent inducers of several cytochrome P450 (CYP) enzymes, most notably CYP1A2, CYP2C9, CYP3A4, and P-glycoprotein (p-gp), leading to a decreased effect of oral anticoagulants and increased ischemic stroke/SEE [41]. Unfortunately, the potential impact of drug interactions was not recorded in this study.

Logistic regression was conducted to identify clinical factors that were associated with a ≥30% decline in the eGFR. This study reported the same findings as those of other research [34,36]. Patient age ≥ 75 years, hypertension, and diabetes mellitus were clinical factors that were associated with the progression of ≥30% decline in eGFR.

## 5. Study Limitations

Several limitations should be considered when interpreting this study’s findings. First, as a retrospective observational study, residual confounding cannot be entirely excluded. Although this study’s team applied a doubly robust approach, combining IPTW with additional multivariable adjustment for covariates that remained imbalanced after weighting (SMD ≥ 0.1) to minimize baseline differences between groups, unmeasured or unknown confounders may still have influenced the results. Second, edoxaban was approved by the Thai Food and Drug Administration (Thai FDA) in December 2016. Thus, the proportion of patients who were treated with edoxaban was small (*n* = 48). This limited sample size may have reduced statistical power and contributed to wider confidence intervals. Accordingly, findings related to the edoxaban subgroup should be considered preliminary and interpreted with caution. Future studies including a larger number of edoxaban users are warranted to validate these observations. Third, the warfarin control quality was suboptimal, with a median TTR of 46.3%, which was lower than that recommended by the ESC guideline for management of AF, which suggests a TTR ≥ 70% for warfarin users. It represents the practical reality of warfarin management in real-world clinical settings. Nevertheless, it should also be acknowledged as a limitation of the study, as suboptimal TTR may favor the outcomes of NOACs. Future studies comparing NOACs with well-controlled warfarin populations (TTR ≥ 70%) are warranted to better determine the magnitude of benefit. Fourth, the potential drug interactions leading to the reduced effect of NOACs and warfarin were not recorded, although this factor may contribute to thrombotic events. Fifth, the ≥30% decline in eGFR was defined using a single measurement without confirmatory repeat testing. Therefore, transient fluctuations in renal function cannot be completely excluded. This reflects the retrospective design and the use of routinely collected clinical data. Future prospective studies with confirmed and sustained eGFR decline are warranted to validate these findings. Sixth, this study detected a difference in the doubling of SCr and AKI for a small number of events. Therefore, further studies are needed to determine whether NOACs provide significant renal benefits in a prolonged duration of follow-up. Finally, although baseline renal laboratory data were complete for all participants, follow-up SCr and eGFR measurements were progressively missing over time at 24 months. This pattern likely reflects irregular laboratory monitoring and loss to follow-up in routine clinical practice. In the time-to-event analyses, patients were censored at the date of their last available laboratory measurement, and Cox proportional hazards models inherently account for right censoring.

## 6. Conclusions

This study provides a safety analysis of NOACs versus warfarin in terms of renal outcomes in real-world practice. NOACs appeared to be associated with a lower risk of adverse renal outcomes, particularly a ≥30% decline in eGFR, than warfarin. In addition, a trend toward a lower risk of ischemic stroke and SEEs was observed in the NOAC group. However, the findings from the subgroup analyses for individual NOACs should be interpreted with caution due to the relatively small sample sizes and limited statistical power. Furthermore, renal outcomes can be influenced by various factors, including co-medication and co-morbidities. Since this study did not directly compare each NOAC head-to-head, the potential impact on renal and clinical outcomes should be considered alongside other clinical factors when selecting an appropriate oral anticoagulant.

## Figures and Tables

**Table 1:** Baseline characteristics of all patients.

Characteristic	Before IPTW			After IPTW		
	NOACs (*n* = 695) *n* (%)	Warfarin (*n* = 761) *n* (%)	*p*-Value	NOACs	Warfarin	SMD
Age (years); median, (IQR)	73 (64, 82)	71 (64, 79)	0.112	70.68 ± 9.32	71.54 ± 12.41	0.078
Sex			0.009			0.166
Male	415 (59.7)	403 (53.0)		48.28	56.57	
Female	280 (40.3)	358 (47.0)		51.72	43.43	
Body weight (kg); median (IQR)	67.5 (58, 77.8)	64 (55, 73)	<0.001	64.80 ± 13.49	66.60 ± 14.42	0.128
BMI (kg/m^2^); median (IQR)	24.9 (22.6, 28)	24.7 (22, 27.5)	0.605	25.01 ± 4.15	25.09 ± 4.68	0.019
LVEF (%); median (IQR)	63.65 (55, 70)	61.8 (50, 68.53)	0.786	58.16 ± 12.95	60.42 ± 12.76	0.175
CHA_2_DS_2_-VASc score; median (IQR)	4 (2, 5)	3 (2, 5)	0.002	3.89 ± 1.64	3.57 ± 1.64	0.189
HAS-BLED score; Median (IQR)	1 (1, 2)	2 (2, 3)	<0.001	2.01 ± 1.06	1.76 ± 1.05	0.236
Comorbidities						
Hypertension	578 (83.2)	641 (84.2)	0.637	88.75	85.15	0.107
Diabetes mellitus	241 (34.7)	256 (33.6)	0.120	34.82	33.68	0.024
Dyslipidemia	465 (66.9)	481 (63.2)	0.073	67.20	64.85	0.050
Coronary artery disease	216 (31.1)	204 (26.8)	0.742	33.86	28.20	0.122
Heart failure	108 (15.5)	206 (27.1)	0.002	29.57	20.87	0.201
Gout	60 (8.6)	72 (9.5)	0.086	6.73	8.10	0.052
Chronic kidney disease	217 (31.2)	263 (34.6)	0.743	25.03	33.19	0.180
Co-medication						
RAAS inhibitors	342 (49.2)	394 (51.8)	0.328	47.78	52.52	0.095
Spironolactone	64 (9.2)	78 (10.2)	0.504	7.06	10.06	0.107
Beta blockers	522 (75.1)	617 (81.1)	0.006	79.63	79.85	0.006
Calcium channel blockers	304 (43.7)	328 (43.1)	0.806	36.11	40.97	0.100
SGLT-2 inhibitors	34 (4.9)	6 (0.8)	<0.001	2.24	1.87	0.026
Statins	526 (75.7)	554 (72.8)	0.209	76.79	74.95	0.043
Antiplatelet agents	204 (29.4)	231 (30.4)	0.676	23.37	26.59	0.074
NSAIDs	20 (2.9)	21 (2.8)	0.892	1.73	2.60	0.060
SBP (mmHg); median (IQR)	130 (117, 139)	130 (118, 140)	0.951	128.64 ± 22.78	130.37 ± 15.41	0.089
Biomarkers); median (IQR)						
Serum Creatinine (mg/dL)	1.01 (0.83, 1.22)	1 (0.80, 1.22)	0.238	0.97 ± 0.34	1.05 ± 0.32	0.270
eGFR (ml/min/1.73 m^2^)	67.19 (55.28, 80.62)	68.12 (53.44, 84.05)	0.414	72.12 ± 20.52	67.32 ± 19.25	0.241
Hb1Ac (%)	5.9 (5.6, 6.6)	6.1 (5.6, 6.9)	0.024	6.18 ± 0.74	6.16 ± 0.83	0.026
LDL (mg/dL)	85 (65.8, 112.4)	88.2 (69, 111.2)	0.321	87.75 ± 26.19	90.94 ± 25.73	0.123
Uric acid (mg/dL)	6.2 (5.1, 7.28)	6.6 (5.4, 8)	0.040	6.36 ± 0.87	6.37 ± 0.77	0.017
TTR (%); median (IQR)	-	46.3 (23.54, 62.37)	-	-	46.3 (23.54, 62.37)	-

Data are presented as median (IQR). Abbreviations: NOACs, non-vitamin K antagonist oral anticoagulants; BMI, body mass index; LVEF, left ventricular ejection fraction; RAAS inhibitors, renin–angiotensin–aldosterone system inhibitors; SGLT-2 inhibitors, sodium-glucose cotransporter-2 inhibitors; NSAIDs, nonsteroidal anti-inflammatory drugs; SBP, systolic blood pressure; eGFR, estimated glomerular filtration rate; HbA1c, hemoglobin A1c; LDL, low-density lipoprotein; TTR, time in therapeutic range; IPTW, Inverse probability of treatment weighted; SMD, standard mean difference.

**Table 2:** Primary outcomes of warfarin versus NOAC users and each NOAC.

Drug (*n*)	≥30% Decline in eGFR *n* (%)	Unadjusted HR (95% CI)	*p*-Value	IPTW-Adjusted aHR (95% CI) *	*p*-Value
Warfarin (761)	143 (18.8)	Reference		Reference	
NOACs (695)	98 (14.1)	0.74 (0.57–0.95)	0.019	0.67 (0.45–1.00)	0.050
Dabigatran (181)	13 (7.2)	0.36 (0.2–0.63)	0.000	0.37 (0.16–0.89)	0.026
Rivaroxaban (249)	43 (17.3)	0.91 (0.65–1.28)	0.595	0.83 (0.50–1.37)	0.456
Apixaban (217)	38 (17.5)	0.94 (0.66–1.35)	0.743	0.78 (0.40–1.51)	0.458
Edoxaban (48)	4 (8.3)	0.42 (0.16–1.13)	0.086	0.32 (0.11–0.89)	0.029

All models satisfied the proportional hazards assumption (global *p* > 0.05). * Adjusted using a doubly robust approach (IPTW plus multivariable adjustment for covariates with SMD > 0.1: sex, body weight, LVEF, CHA_2_DS_2_-VASc, HAS-BLED, hypertension, coronary artery disease, heart failure, chronic kidney disease, calcium channel blocker, spironolactone, SCr, eGFR, and LDL). Abbreviations: NOACs, non-vitamin antagonist oral anticoagulants; eGFR, estimated glomerular filtration rate; aHR, adjusted hazard ratio; 95% CI, 95% confidence interval.

**Table 3:** Doubling of SCr and AKI in warfarin group versus NOAC group.

Drug (*n*)	Number of Patients *n* (%)	Unadjusted HR (95% CI)	*p*-Value	IPTW-Adjusted aHR (95% CI) *	*p*-Value
Doubling of SCr ^a^					
Warfarin (761)	16 (2.1)	Reference		Reference	
NOACs (695)	12 (1.7)	0.82 (0.39–1.73)	0.605	0.64 (0.24–1.72)	0.373
Rivaroxaban (249)	6 (2.4)	1.15 (0.49–2.93)	0.774	1.16 (0.41–3.33)	0.776
Apixaban (217)	6 (2.8)	1.32 (0.52–3.78)	0.559	0.77 (0.22–2.66)	0.674
Acute kidney injury ^b^					
Warfarin (761)	81 (10.6)	Reference		Reference	
NOACs (695)	58 (8.3)	0.78 (0.56–1.09)	0.147	0.69 (0.41–1.17)	0.169
Dabigatran (181)	8 (4.4)	0.40 (0.19–0.83)	0.014	0.61 (0.20–1.86)	0.389
Rivaroxaban (249)	24 (9.6)	0.90 (0.57–1.43)	0.663	0.63 (0.32–1.26)	0.195
Apixaban (217)	24 (11.1)	1.05 (0.67–1.66)	0.827	0.93 (0.39–2.20)	0.869
Edoxaban (48)	2 (4.2)	0.39 (0.09–1.57)	0.184	0.27 (0.06–1.17)	0.080

* Adjusted using a doubly robust approach (IPTW plus multivariable adjustment for covariates with SMD > 0.1: sex, body weight, LVEF, CHA_2_DS_2_-VASc, HAS-BLED, hypertension, coronary artery disease, heart failure, chronic kidney disease, calcium channel blocker, spironolactone, SCr, eGFR, and LDL. ^a^ This model satisfied the proportional hazards assumption (global *p* > 0.05). ^b^ This model did not satisfy the proportional hazards assumption (global *p* < 0.05). Abbreviations: SCr, serum creatinine; IPTW, inverse probability of treatment weighted; NOACs, non-vitamin antagonist oral anticoagulants; aHR, adjusted hazard ratio; 95% CI, 95% confidence interval.

**Table 4:** Incidence rates of stroke and systemic embolism.

Drug (*n*)	Number of Patients *n* (%)	Incidence Rate (Per 100 Person-Year)	Unadjusted HR (95% CI)	*p*-Value	IPTW-Adjusted aHR (95% CI) *	*p*-Value
Warfarin (761)	36 (4.73)	3.02	Reference		Reference	
NOACs (695)	13 (1.87)	1.15	0.45 (0.24–0.85)	0.015	0.49 (0.22–1.10)	0.084

* Adjusted using a doubly robust approach (IPTW plus multivariable adjustment for covariates with SMD > 0.1: sex, body weight, LVEF, CHA_2_DS_2_-VASc, HAS-BLED, hypertension, coronary artery disease, heart failure, chronic kidney disease, calcium channel blocker, spironolactone, SCr, eGFR, and LDL. All models satisfied the proportional hazards assumption (global *p* > 0.05). Abbreviations: NOACs, non-vitamin antagonist oral anticoagulants; aHR, adjusted hazard ratio; 95% CI, 95% confidence interval.

**Table 5:** Independent predictors of the ≥30% decline in the eGFR.

Variables	OR (95% CI)	*p*-Value
NOACs	0.70 (0.52–0.93)	0.014
Female	1.17 (0.86–1.58)	0.317
Age ≥ 75 years	1.72 (1.28–2.32)	<0.001
Body weight ≤ 60 kg	0.84 (0.62–1.16)	0.289
Hypertension	1.82 (1.11–2.99)	0.018
Diabetes mellitus	1.59 (1.18–2.13)	0.002

Abbreviations: NOACs, non-vitamin antagonist oral anticoagulants; eGFR, estimated glomerular filtration rate; OR, odds ratio; 95% CI, 95% confidence interval.

## Data Availability

The datasets used and/or analyzed during the current study are available from the corresponding author upon reasonable request.

## Abbreviations

The following abbreviations are used in this manuscript:

AF	atrial fibrillation
AKI	acute kidney injury
BMI	body mass index
CAD	coronary artery disease
CKD	chronic kidney disease
CKD-EPI	Chronic Kidney Disease Epidemiology Collaboration
eGFR	estimated glomerular filtration rate
ESRD	end-stage renal disease
HbA1c	hemoglobin A1c
INR	international normalized ratio
LDL	low-density lipoprotein
LVEF	left ventricular ejection fraction
NOACs	non-vitamin K antagonist oral anticoagulants
NSAIDs	nonsteroidal anti-inflammatory drugs
NVAF	nonvalvular atrial fibrillation
PAR	protease-activated receptor
RAAS inhibitors	renin–angiotensin–aldosterone system inhibitors
SBP	systolic blood pressure
SCr	serum creatinine
SEE	systemic embolism event
SGLT-2 inhibitors	sodium-glucose cotransporter-2 inhibitors
TTR	time in therapeutic range
VKAs	vitamin K antagonists
WRN	warfarin-related nephropathy
